# Computational detection and quantification of human and mouse neutrophil extracellular traps in flow cytometry and confocal microscopy

**DOI:** 10.1038/s41598-017-18099-y

**Published:** 2017-12-19

**Authors:** Brandon G. Ginley, Tiffany Emmons, Brendon Lutnick, Constantin F. Urban, Brahm H. Segal, Pinaki Sarder

**Affiliations:** 1 Department of Pathology & Anatomical Sciences, SUNY Buffalo, USA; 2 Department of Immunology, Roswell Park Cancer Institute (RPCI), Buffalo, USA; 3 Department of Clinical Microbiology and Umeå Centre for Microbial Research, Umeå University, Umeå, Sweden; 4 Department of Medicine, Roswell Park Cancer Institute (RPCI), Buffalo, USA; 5 Department of Medicine, SUNY Buffalo, USA; 6 Department of Biomedical Engineering, SUNY Buffalo, USA; 7 Department of Biostatistics, SUNY Buffalo, USA

## Abstract

Neutrophil extracellular traps (NETs) are extracellular defense mechanisms used by neutrophils, where chromatin is expelled together with histones and granular/cytoplasmic proteins. They have become an immunology hotspot, implicated in infections, but also in a diverse array of diseases such as systemic lupus erythematosus, diabetes, and cancer. However, the precise assessment of *in vivo* relevance in different disease settings has been hampered by limited tools to quantify occurrence of extracellular traps in experimental models and human samples. To expedite progress towards improved quantitative tools, we have developed computational pipelines to identify extracellular traps from an *in vitro* human samples visualized using the ImageStream^®^ platform (Millipore Sigma, Darmstadt, Germany), and confocal images of an *in vivo* mouse disease model of *aspergillus fumigatus* pneumonia. Our two *in vitro* methods, tested on *n* = 363/*n* =145 images respectively, achieved holdout sensitivity/specificity 0.98/0.93 and 1/0.92. Our unsupervised method for thin lung tissue sections in murine fungal pneumonia achieved sensitivity/specificity 0.99/0.98 in *n* = 14 images. Our supervised method for thin lung tissue classified NETs with sensitivity/specificity 0.86/0.90. We expect that our approach will be of value for researchers, and have application in infectious and inflammatory diseases.

## Introduction

Neutrophils are phagocytes that envelope and digest microbes and other foreign objects for elimination^1^. Generation of neutrophil extracellular traps (NETs) is a distinct mode of cell death that targets extracellular pathogens^2^. Molecularly, NETs are a complex of processed chromatin bound to granular and cytoplasmic proteins which is expelled from the cell onto pathogens^3^. Although NETs play a role in trapping and killing extracellular pathogens, thereby preventing dissemination, NETs are injurious and considered to play a role in a wide array of inflammatory diseases, such as, acute respiratory distress syndrome^4^, systemic lupus erythematosus^3^, rheumatoid arthritis^5^, sepsis^3,6–8^, diabetes^9^, and cancer^10–12^. The exact contribution of NETs to clearance of pathogens versus inflammatory injury is less well understood. Deeper exploration into the mechanisms that drive NET formation is required to evaluate the immunological impact of NET formation, the local cellular implications of NET formation, the use of NETs as a predictive biomarker for various diseases, and targeting NETs therapeutically.

Further understanding of NETs and their implications will be hampered if researchers are confined to the realm of manual quantification. The lack of a rigorous digital microscope-to-quantification protocol for automatically quantifying NETs inhibits their use as prognostic biomarkers and therapeutic targets. There are two semi-automatic approaches developed for *in vitro* NET estimation that involve quantifying the morphological spatial distribution of NET constituents^13,14^, and two fully automated approaches to estimate NETs by neutrophil morphology^15,16^. Clearly a fully automated software is more optimal than semi-automated and flow cytometry is ideal for rapid and objective quantification of NETs, but doesn’t have the capacity for cellular imaging, and can’t be applied to quantification of NETs in tissue. Gavillet *et al*.^14^ have designed a flow cytometric assay for quantification of NETs in blood using antibodies against NET constituents, DNA, modified histones, and granular enzymes. The ImageStream^®^ platform (Millipore Sigma, Darmstadt, Germany), which combines flow cytometry based quantitation with cellular imaging, is an ideal modality for tackling this problem; a large population of neutrophils can be stimulated with agents of interest and each neutrophil can be imaged one by one to see if it has produced a NET. Then, aggregate conclusions about the neutrophil population can be drawn. The last obstacle, then, is the development of a fully automated image analysis software capable of identifying NETs.

Toward this end, we have developed a computational method capable of NET classification and quantification by imaging of neutrophil DNA. NETs were defined by the morphological presence of extracellular DNA from purified neutrophils. The objects in both classes are distinct enough that a support vector machine (SVM)^17^ is able to efficiently discriminate the objects with high performance. We have also implemented an alternative convolutional neural network (CNN)^18^ approach for binary image classification. Our long-term goal is to apply these methods to rapidly quantify the response of neutrophils to infection and injury.

Regarding identification of *in vitro* images of human NETs, our SVM method, trained on *n* = 1092 images and tested on a holdout set of *n* = 363 images, classified NETs vs intact neutrophils with 0.98 sensitivity and 0.93 specificity. Alternatively, the CNN method achieved 95.5% validation accuracy, trained on *n* = 908 images, augmented by rotation to *n* = 6414. When tasked to classify *n* = 145 holdout images, the network classified NETs against neutrophils with 1 sensitivity and 0.92 specificity.

Our second goal was to investigate computational methods for identifying NETs in *in vivo* tissue sections of pulmonary infection, and compare performance to the gold standard, NET identification by visual inspection of immunofluorescence microscopy. In our earlier work, we found that the phagocyte NADPH oxidase was required for NET generation during murine pulmonary aspergillosis^19^. The approach involved pulmonary challenge with *A*. *fumigatus* hyphae, followed by quantification of airway and alveolar neutrophilic inflammation and NETosis through immunofluorescence staining of NET constituents (e.g., DNA, histones, and the granular constituent, myeloperoxidase (MPO)) visualized with confocal microscopy. Using lung sections from these experiments, we have developed an unsupervised computational pipeline exploiting the inherent co-localization of histone, DNA, and MPO within NETs. Briefly, an unsupervised classification criterion for each fluorescent histone object is derived as the percent of pixels within its area that have decondensed nuclear material colocalized with MPO. An alternative supervised approach to classification can be attained by extracting the colocalization data used for the unsupervised method and classifying objects by deep CNN. Regarding classification performance in thin tissues, for *n* *=* 14 images, the unsupervised method scored pixel-wise sensitivity/specificity 0.99/0.98. The CNN method, which operates on object patches rather than entire images, scored object-wise holdout sensitivity and specificity of 0.86 and 0.90 on *n* = 631 object patches derived from a holdout set of 2 images. Together, these studies support the future application of computational imaging for objective and rapid identification of NETs in various inflammatory diseases.

## Results

### Computational pipeline overview

We have developed four computational pipelines to automatically estimate NETs, two for ImageStream^®^ images of *in vitro* stimulated healthy donor neutrophils and two for analysis of lung sections from murine pulmonary aspergillosis.

### Automated quantification of human NETs

**Figure 1:**
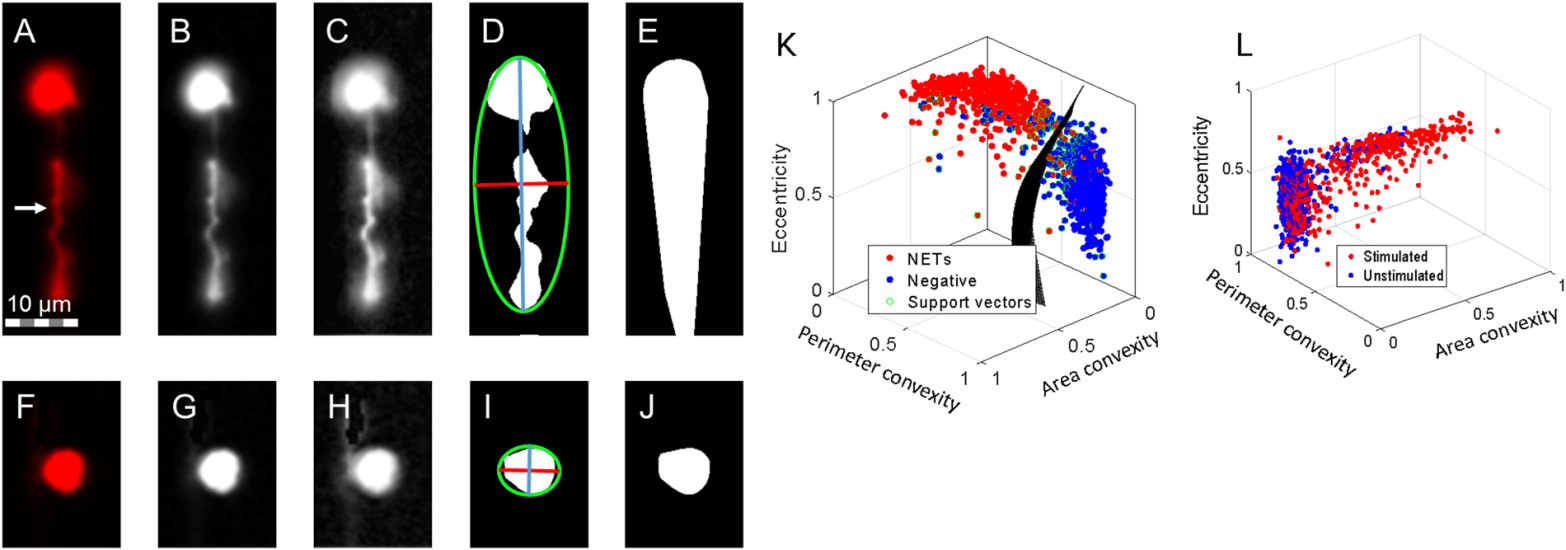
Computational pipeline to identify *in vitro* neutrophil extracellular traps (NETs) with morphology. (**A–E**) are NET-positive and (**F–J**) are NET-negative. (**A** & **F**) Respective raw examples of a NET object and non-NET object. NET indicated with white arrow in (**A**). (**B** & **G**) Grayscale versions of the preceding images. (**C** & **H**) Enhanced contrast using contrast limited adaptive histogram equalization. (**D** & **I**) Ellipse fitting of the binary region, minor and major axes are demonstrated. (**E** & **J**) Convex hull fitting of binary regions. (**K**) Distribution of the three most separable morphological features for both image classes, displaying the optimal support vector hyperplane for *n* = 1455 images. (**L**) Morphological distributions for *n* = 568 unstimulated neutrophils and *n* = 294 stimulated neutrophils from an independent experiment.

We have developed a pipeline to computationally, automatically discriminate NETs from non-NETotic neutrophils in ImageStream^®^ images of healthy donor neutrophils stimulated with phorbol myristate acetate to induce NETs^20^. Unstimulated neutrophils were used as controls. This specific processing task is well suited for morphological quantification, as the objects are simple geometric shapes. Figure 1 graphically exhibits the simple pipeline to extract meaningful morphological features of *in vitro* NETs. Figure 1A–E exemplify a positive case (NET), and Fig. 1F–J exemplify a negative case (intact neutrophil). NET DNA trail, stained by DRAQ5 (see *In vitro* data preparation), is identified in Fig. 1A with a white arrow. Some DNA trails had dim intensity; therefore, the first step is to enhance contrast with contrast limited adaptive histogram equalization (CLAHE). Six morphological features were derived as discussed in *in vitro* computational pipeline section of the methods, and Table 1 presents the resulting distributions. Convex area, area convexity (ratio between the respective object area and its convex hull^21^ area), and perimeter convexity (ratio between the respective object perimeter and its convex hull perimeter) were selected because the NETs are highly concave, and intact neutrophils are highly convex. Further, we found that the morphological structures of NETs in this experiment tend to be long and thin, whereas intact neutrophils tend to be morphologically rounded and circular. These characteristics justify using object eccentricity and ratio between the object’s equivalent diameter and minor axis length as features as well. Mean intensity was selected because NET chromatin trails, at first, appeared to be dimmer than their neutrophil counterparts. The top three optimal features were determined using *rankfeatures* from MATLAB, using an absolute value two-sample t-test with pooled variance estimate as rank criteria. Fig. 1K shows an example hyperplane trained on the most separable three features for *n* = 1455 images, where *n* = 928 were negative and *n* = 527 were positive. The three other features did not provide unique information about the top three or provided no improvement to classification accuracy (see Fig. S3). The hyperplane in Fig. 1K was trained with a Gaussian kernel σ = 2 for the image resolution defined in the Methods section, resulting in training sensitivity/specificity 0.96 0.92. Conversely, when trained on *n* = 1092 images and tested on a random holdout set of *n* = 363 images, we obtained holdout 0.98 sensitivity and 0.93 specificity. For comparison, Fig. 1L shows the distribution of morphological features for one data set, 568 unstimulated neutrophils versus another set of 294 stimulated neutrophils.

**Table 1:** Features of NET-positive and -negative neutrophils.

	Positive	Negative
Convex area (µm^2^)	199 ± 64	89 ± 13
Mean intensity (a.u.)	0.05 ± 0.01	0.042 ± 0.01
Area convexity	0.24 ± 0.07	0.04 ± 0.01
Perimeter convexity	0.37 ± 0.08	0.68 ± 0.02
Eccentricity	0.82 ± 0.05	0.49 ± 0.01
Equivalent diameter/minor axis length	1.45 ± 0.11	1.09 ± 0.004

### Automated quantification of NETs in thin tissues

**Figure 2:**
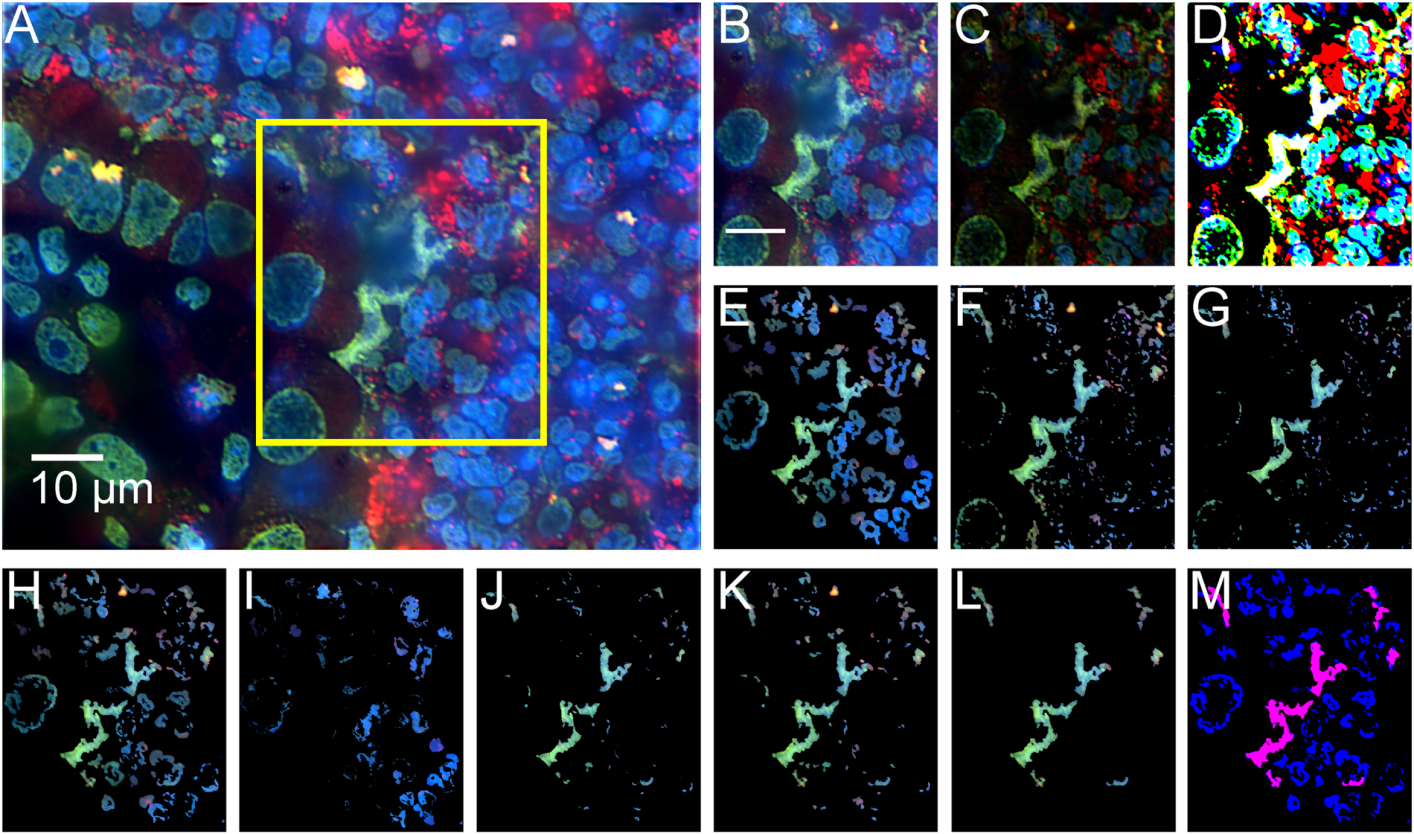
Unsupervised pipeline to identify neutrophil extracellular traps (NETs) from immunofluorescence images of mouse lung following Aspergillus fumigatus infection. (**A**) Raw image. DNA is identified by DAPI staining (blue) and primary antibodies directed against MPO (red) and histone H1 (green) were detected with AlexaFluor 568- and 488- conjugated secondary antibodies. Yellow box indicates sub-region for **B**–**M**. (**B**) Sub-image of **A**. (**C**) Image shown in **B** after top-hat filtering. (**D**) Visualization of the image after normalization. (**E**) Bradley local thresholding defines a master object mask. (**F**) Objects with co-localized levels of histoneand myeloperoxidaseboth greater than one unit of intensity standard deviation in the respective channels. (**G**) Objects with all markers co-localized. (**H**) Objects where histone marker intensity is greater than DNA marker. (**I**) Objects where DNA marker exhibits higher intensity than histone marker. (**J**) Intersection of the images in **F**,**G**, and **H**, with pixels contained in I set to zero. (**K**) Reconstruction of the image shown in **J** underneath the image in F, followed by morphological noise removal in (**L**). (**M**) Visualization of the extracted co-localized regions (pink) over the master object regions (blue).

**Figure 3:**
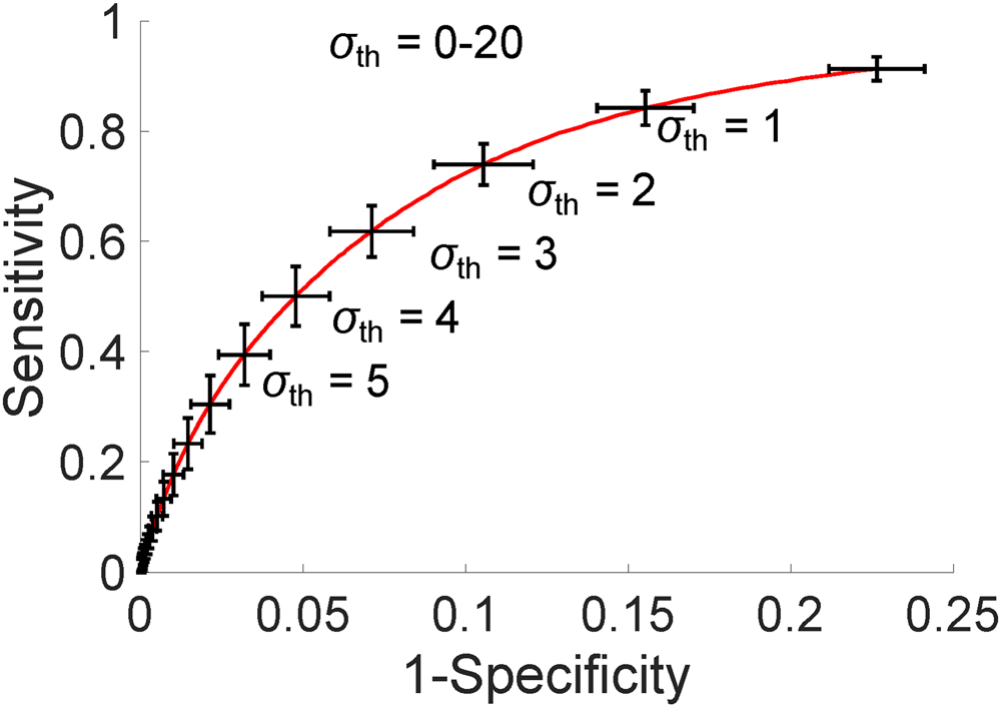
Receiver operating characteristic of NETs identification based on co-localized MPO and histone with respective intensity levels above a multiple of the intensity standard deviations (${\sigma }_{{\rm{th}}}$) in the respective channels. Curve represents mean sensitivity and specificity for *n* = 14 images. Error bars represent standard deviations in the respective directions. The thresholds ${\sigma }_{{\rm{th}}}$ are sampled by steps of 0.01, in a range of 0-20, and are labeled for five demonstration points on the curve.

**Figure 4:**
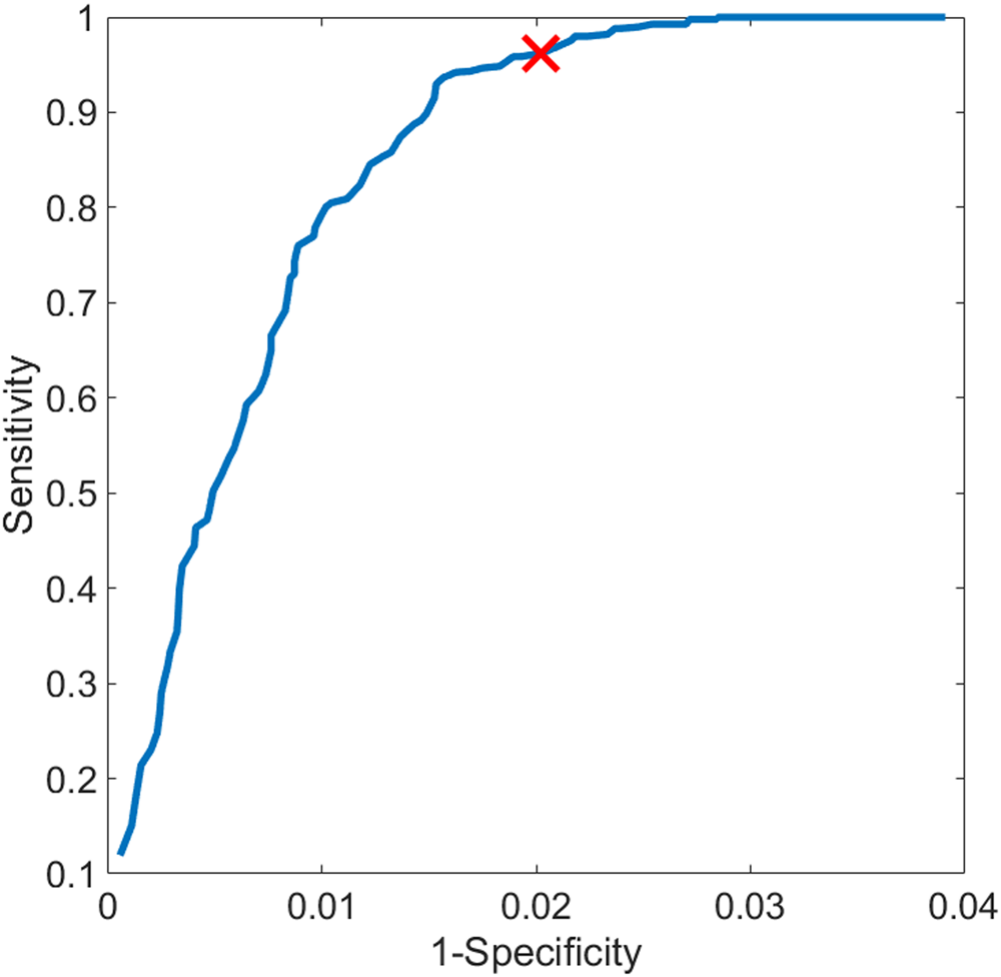
Receiver operating characteristic curve for neutrophil extracellular traps (NETs) classification. We are using co-localization threshold for *n* = 14 images with *n* = 527 NET objects and *n* = 2808 negative objects. Sensitivity and specificity are recorded pixel-wise. The red cross reports a mean pixel-wise sensitivity/specificity of 0.99/0.98.

Wild type and NADPH oxidase-deficient mice were administered *A. fumigatus* hyphae, sacrificed, and their lungs were harvested for assessment of NETs. These digital images were collected as part of the study conducted by Rohm *et al*.^19^. Figure 2 visually demonstrates the objects within each image at each step of the NET extraction. The overall idea behind the method is to use simple morphology of co-localized markers of NETs to guide decision making and sequentially eliminate pixels which do not fit the correct biological criteria (for example, NETs must contain some amount of histone^3^). Raw images, such as the one shown in Fig. 2A, are top-hat processed to remove uneven illumination. (Fig. 2B is a sub-region of Fig. 2A for demonstration, marked with a yellow box.) Fig. 2C shows image after top-hat processing. Next, all pixels in the image are normalized by the global mean and standard deviation of pixel intensities, shown in Fig. 2D. Figure 2E shows Bradley local thresholding^22^ of normalized histone channel, which is used to define foreground from background within a local sliding window. This creates a *master* mask defining which pixels are object and which are background. The next object mask locates pixels which have value greater than one-unit standard deviation in intensity above the mean in both the MPO channel and the histone channel (Fig. 2F). Our observation was that the histone and MPO components of NETs are almost always greater than one standard deviations brighter than their surroundings, but the DNA component was not. As justification, Fig. 3 demonstrates a receiver operating characteristic curve for NETs identification using the multiples of the standard deviation of the respective histone and MPO intensity levels as thresholds (${\sigma }_{{\rm{th}}}$). Here the co-localized objects formed by the thresholded histone and MPO channel images identify the NETs. In this curve, each point specifies a step of ${\sigma }_{{\rm{th}}}$ = 0.01 on the range [0,20], and details the mean sensitivity and specificity in identifying NETs, averaged for *n* = 14 images, compared against our ground truth annotations. Error bars represent the standard deviation of that metric in the respective direction. A threshold value corresponding to one-unit intensity standard deviation provides 0.84/0.85 sensitivity/specificity, which, in turn, provides sensitivity/specificity 0.99/0.98 in our final NETs identification (see below), and thus offers a desired performance with > 0.95 sensitivity/specificity in detection. Because NETs must contain DNA, another mask identifies objects which have high levels of co-localized histone and MPO, co-localized with any amount of DNA (Fig. 2G). The next step identifies pixels that exhibit greater histone intensity than DNA intensity (Fig. 2H), suggesting they are decondensed. Conversely, objects which have higher DNA intensity than histone intensity are set to zero, such as those in Fig. 2I. The intersection of the masks in Fig. 2F–H is obtained (shown Fig. 2J), and flood-filled^23^ under the image shown in Fig. 2F to preserve morphological structure (result in Fig. 2K). Figure 2L shows objects smaller than 2 µm^2^ being removed from Fig. 2K. We termed this mask as the *sub-master* mask, and is a pseudo-NET criterion when evaluated with respect to full histone objects identified in the *master* mask (Fig. 2M, the percentage of pink area within a blue area; note here the blue areas are hidden underneath the respective pink areas). The area of this *sub-master* mask, taken as a percentage of the area of histone (*master*) in which it is contained, serves to be an unsupervised marker of NETs. The ratio corresponding to this percentage is defined as the *co-localization level*, which is compared with a *co-localization threshold* for NETs detection. Figure 4 shows the receiver operating curve when using this *co-localized threshold* for unsupervised NET detection (averaged over *n* = 14 images). A red colored cross marks the point of highest sensitivity, 0.96, while keeping specificity fixed at 0.98. These sensitivities and specificities are calculated pixel-wise. Specificity under this method, assuming a similar imaging setup and acquisition, should generally score high; there are a large amount of objects in the fluorescence image which contain no co-localization of all the markers, allowing one to dramatically reduce the number of objects (under the assumption NETs are a minority class of the image).

### Alternate convolutional neural network method

We trained two deep CNNs to demonstrate feasibility and performance as an alternative method for binary classification of NETs in both *in vitro* and *in vivo* thin tissue section images of NETs. Our deep CNNs employ GoogLeNet architecture; see Methods for the information on training parameters. The *in vitro* CNN was fed with images similar to raw data in Fig. 1A and F, upon assigning regions with lower 10% intensity levels in the respective images as background. The dataset of *n* *=* 1,455 images was partitioned into *n* = 908/402/145 images for training/validation/holdout testing, respectively. The data was augmented to increase the number of training samples by rotating images on seven fixed angles between 45° and 315°, in intervals of 45°. Data augmentation improves the spatial invariance and the performance of the deep CNN model^24^. This resulted in a total of *n* *=* 6,414 training samples. The network achieved 95.5% accuracy and 0.12 loss on the validation data upon convergence. Validation loss refers to the total sum of errors made by the network on a holdout dataset, and therefore is ideally as low as possible^25^. On *n* = 145 images that the network was not trained on, the network labeled images with 1 sensitivity and 0.92 specificity.

To eliminate the *co-localization threshold* parameter and to develop fully automatic method for the thin tissue section images, we used deep CNN for detecting highly heterogeneous NETs structure from the lung section images. This *in vivo* CNN for thin tissue sections was trained with pseudo RGB images, which were composed using *master* and *sub-master* objects as discussed in the unsupervised method above, plus the raw data. The first channel of such RGB image is the binary version of the *sub-master* object image. The second is the normalized intensity of the corresponding *master* object image. The third is the binary version of regions of co-localized MPO and histone, with respective intensities greater than one-unit intensity standard deviation of the respective channels, where histone level is greater than the DNA level. The third channel considers the same region corresponding to the *master*/*sub-master* region in the first two channels. Supervision is done using the ground-truth levels based on *master* objects. To increase the training sample size, the dataset was augmented based on the same rotation angles as used for the *in vitro* case. Because the sample size in the NET object class was imbalanced with that of the negative object class (527 vs 2808), the data of the minority class (NETs) was sampled disproportionately to the majority class (non-NETs, i.e., intact cells of mucosal and submucosal layers, fluorescent noise, or intact neutrophils) following established methods^26^ during the data augmentation. A random multiplier between 0 and 1 was applied to each rotation angle during the augmentation while sampling each NET object for five times as often as a non-NET object. For performance evaluation, the *in vivo n* *=* 14 thin tissue images were first partitioned globally by image, resulting in *n* = 8/4/2 thin tissue images for training/validation/holdout testing. From these images, all NET or non-NET objects were extracted, resulting in *n* = 1735/969/631 object patches for training/validation/holdout testing, respectively. Data augmentation created *n* *=* 19430 training objects. The network for *in vivo* binary NET classification achieved 94.5% accuracy and 0.13 loss on the validation data upon convergence. On the *n* = 631 objects taken from *n* *=* 2 holdout test images, the CNN classified NETs with 0.86/0.9 sensitivity/specificity. Performance here is expected to improve with a larger training set. These results are summarized in Table 2.

**Table 2:** Training data and performance of CNN approach.

	*In vitro* images	*In vivo* object patches
Training data	908 (6414)	1735 (19430)
Validation data	402	969
Holdout data	145	631
Validation accuracy (%)	95.5	94.5
Validation loss	0.12	0.13
Holdout sensitivity	1	0.86
Holdout specificity	0.92	0.90

### Comparison of methods

We compared the performance of the SVM morphology method and the CNN method for *in vitro* NET identification. Table 3 compares the predictions of both classifiers. Two of the datasets were stimulated with PMA and one dataset was an unstimulated control (the unstimulated control still displays a small of NET generation, though mostly negative). The CNN tended to classify more NETs than human annotators. Overall the SVM method provided much higher performance. Because our presented methods for mouse lung sections are inherently different (supervised vs unsupervised), we did not perform a detailed comparison between the two.

**Table 3:** Comparison of performance by SVM and deep CNN.

		Ground-truth	SVM	Deep CNN
Stimulated set 1	NET	174	165	191
	Negative	120	129	103
Stimulated set 2	NET	314	311	370
	Negative	279	282	223
Unstimulated	NET	75	59	105
	Negative	493	493	463
Overall	NET	563	535	666
	Negative	892	920	789
Sensitivity	------------	1	0.96	0.84
Specificity	------------	1	0.92	0.88

Coelho *et al*.^16^ developed a method for *in vitro* NETs identification. This method works in three general steps. In the first step, the image is partitioned into discrete rectangular regions. The second step involves learning a regression trend between computationally quantified features from the objects in such regions with manually predicted amount of NETs in the region. In the third step, a linear adjustment is applied to correct for biases to derive a characteristic curve to classify a region or the pixel at its center as NET or non-NET. Two major differences between this method and our method are: (i) we have expanded NETs analysis to include solutions for both *in vitro* images and *in vivo* thin tissue section images; and (ii) the unsupervised version of our method does not require training, thus requires significantly less time.

**Figure 5:**
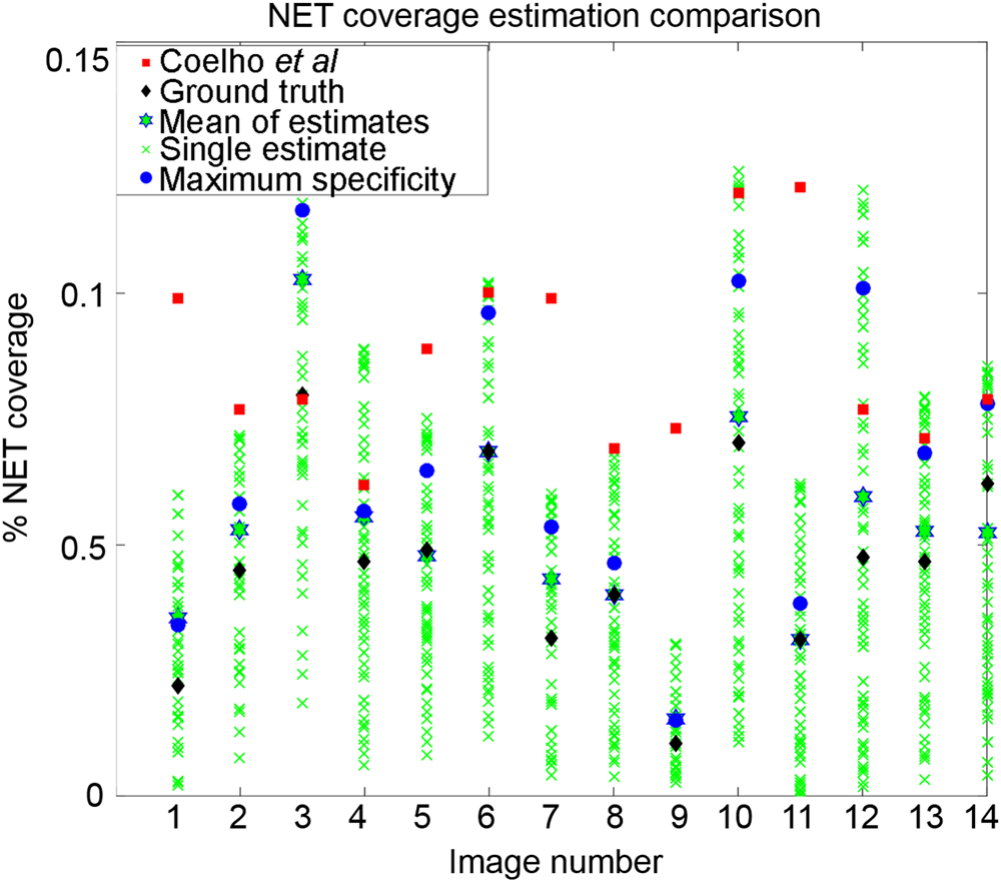
Comparison of unsupervised NETs segmentation from thin tissue sections against the Coelho’s method. Green crosses represent various co-localization thresholds between 0 and 1 and the corresponding NET coverage estimate. Black diamonds signify manually annotated ground-truths. Red boxes demonstrate estimates made by the Coelho’s method. Green stars with blue outlines identify the mean coverage estimate at all thresholds (see IMNCE in Methods), which appears to be a reliable estimator for NET coverage. Blue circles identify the threshold value which achieves the highest sensitivity and specificity.

Figure 5 demonstrates a comparison between our unsupervised method for labeling NETs in thin tissues and the supervised method developed by Coelho *et al*.^16^. To compare fairly, we retrained their entire model on our data. Each green cross in the plot represents the percent *neutrophil extracellular trap (NET) coverage* estimation of our method at each *co-localization threshold*. NET coverage refers to the percentage of pixels of the input image that are classified as NETs. The black diamonds represent the ground-truth NET coverage estimates. Blue dots show the threshold of our method which achieved maximum sensitivity while holding specificity fixed at 0.98, for each image. Red squares indicate the estimation of NET coverage by Coelho’s. method. Interestingly, taking the mean of all NET coverage (IMNCE; see Methods) estimates from all thresholds on the interval (0, 1] yields a new estimator which is quite close to the ground truth NET coverage; further, it even intersects with the ground truth in several images. IMNCE is shown in Fig. 5 as a green star with blue outline. Our proposed method requires only ~15.7 s to estimate NETs in one of our images using a computer with an Intel Core i7-4790 and 8 Gb RAM. Conversely, the Coelho’s method required 560 s for each of our images.

## Discussion

Our results demonstrate the feasibility of a rapid computational approach for NET quantification in stimulated circulating human neutrophils and in mouse lung sections during pneumonia. For *in vitro* classification, the SVM provided a more well-rounded performance across multiple separate datasets than the CNN. This is intuitive by their design, since SVM methods do not require gratuitous amounts of training data, where for some tasks neural networks may need thousands of unique examples to approach optimal performance. We also demonstrated the feasibility of the CNN approach for NET identification in lungs during murine pulmonary aspergillosis. The current CNN was trained using a relatively low number of NET objects relative to negative objects (527 positive vs 2808 negative), and further, the fluorescence co-localization of bronchial epithelial cells was found to be similar to NETs (decondensed, with sparse MPO colocalization) with a different morphological distribution, (see Fig. S2H). To mitigate the class imbalance, our positive dataset was sampled for more times during the data augmentation than the negative to balance the classes^26^. However, it is always desirable to have additional real data rather than additional synthetic data, and, as such, having a larger database of NET objects in the future will increase the sensitivity of the CNN approach, because CNNs require high number of training samples to achieve optimal performance. Further examples of typical NET objects can be found in supplementary Figs. S1 & S2. Our future work will look to expand our image sets to be larger and more diverse for a number of inflammatory diseases that drive NET generation (e.g., sepsis and vasculitis).

Our analysis is replicable under the constraint of a similar imaging system, resolution, and computational parameter settings as those used in this work. Note that our single cell studies involved stimulation of normal donor neutrophils with PMA, a potent inducer of NET generation. PMA is a non-physiological stimulus, and it will be important to evaluate our computational approaches on neutrophils from patients with diseases associated with NETosis (e.g., sepsis). Another limitation is that pulmonary aspergillosis induces an exuberant neutrophilic inflammatory response; the performance of our approach may be affected by different disease models and by lower levels of neutrophilic inflammation.

Computational, high throughput identification of NETs in circulating neutrophils is expected to create standardized protocols for quantifying NETosis during inflammatory diseases, such as sepsis and vasculitis, and may lead to novel prognostic biomarkers. In addition, automatic identification of NETs from thin tissue sections is expected to expedite analysis of experimental models involving neutrophilic inflammation and injury and the effect of therapies.

## Methods

All of the processing discussed below, aside from CNN training, was performed using MATLAB (MathWorks, Natick, MA).

### Ground truth annotation

In murine aspergillosis studies, ground truth masks were obtained by co-authors Drs. Brahm Segal and Constantin F. Urban based on visual inspection of immunofluorescent images, which is the current gold standard for NET identification. Figure 2 includes unpublished images from Rohm *et al*.^19^.

### Imaging resolution and parameters

Table 4 details imaging specifics and computational parameters used in this study.

**Table 4:** Imaging systems, resolutions, and computational parameters.

In vitro	
Image system	ImageStream^®^
Pixel resolution	0.17 µm
SVM kernel $\sigma$	2
CLAHE $\alpha$	0.01
Thin tissue section	
Image system	Nikon Eclipse Confocal C1
Pixel resolution	0.07 µm
*σ*_H_	1
*σ*_M_	1
Area minimum threshold	2 µm^2^
Co-localization threshold	0.32
Top-hat disk radius	1.67 µm
Bradley threshold	1%
Bradley window	3.3 × 3.3 µm^2^
Morphological cleaning disk radius	0.2 µm
Morphological cleaning lines	0.3 µm

### *In vitro* data preparation

Neutrophils were isolated from healthy human donor blood and isolated using Histopaque-based density gradient centrifugation described in Swamydas *et al*.^27^. Briefly, 8 ml of blood was layered over Histopaque 1077 and Histopaque 1119, and centrifuged for 30 min at 500 g without brake. Neutrophils were settled between the Histopaque 1077 and Histopaque 1119 interface. Neutrophils were collected and stimulated for 2 h with 20 ng/ml phorbol myristate acetate (PMA; Sigma-Aldrich, St. Louis, MO) as a positive control for NET generation. Polystyrene tubes were used. PMA stimulation was stopped by washing cells in PBS. Cells were FC blocked and stained with FITC conjugated anti-CD15 (eBioscience, San Diego, CA) for 30 minutes at room temperature. Cells were washed and resuspended in 50 μl PBS. Samples were transferred to 1.5 ml eppendorf tubes and stained with 5 μm DRAQ5 (ex/em (nm): 681/697; Thermo Fisher Scientific, Waltham, MA) right before analysis on ImageStream®. Note that we use the DRAQ5 fluorescence images in this work for our computational analysis. We followed a protocol approved by Institutional Review Board at Roswell Park Cancer Institute, and informed consent from study participant was obtained. All methods were performed in accordance with the relevant federal guidelines and regulations. A typical NET shape varies from 7–20 µm along the major axis and 1-5 µm along the minor axis. A typical neutrophil nucleus shape is circular with diameter 6-7 µm.

### *In vitro* computational pipeline

**Figure 6:**
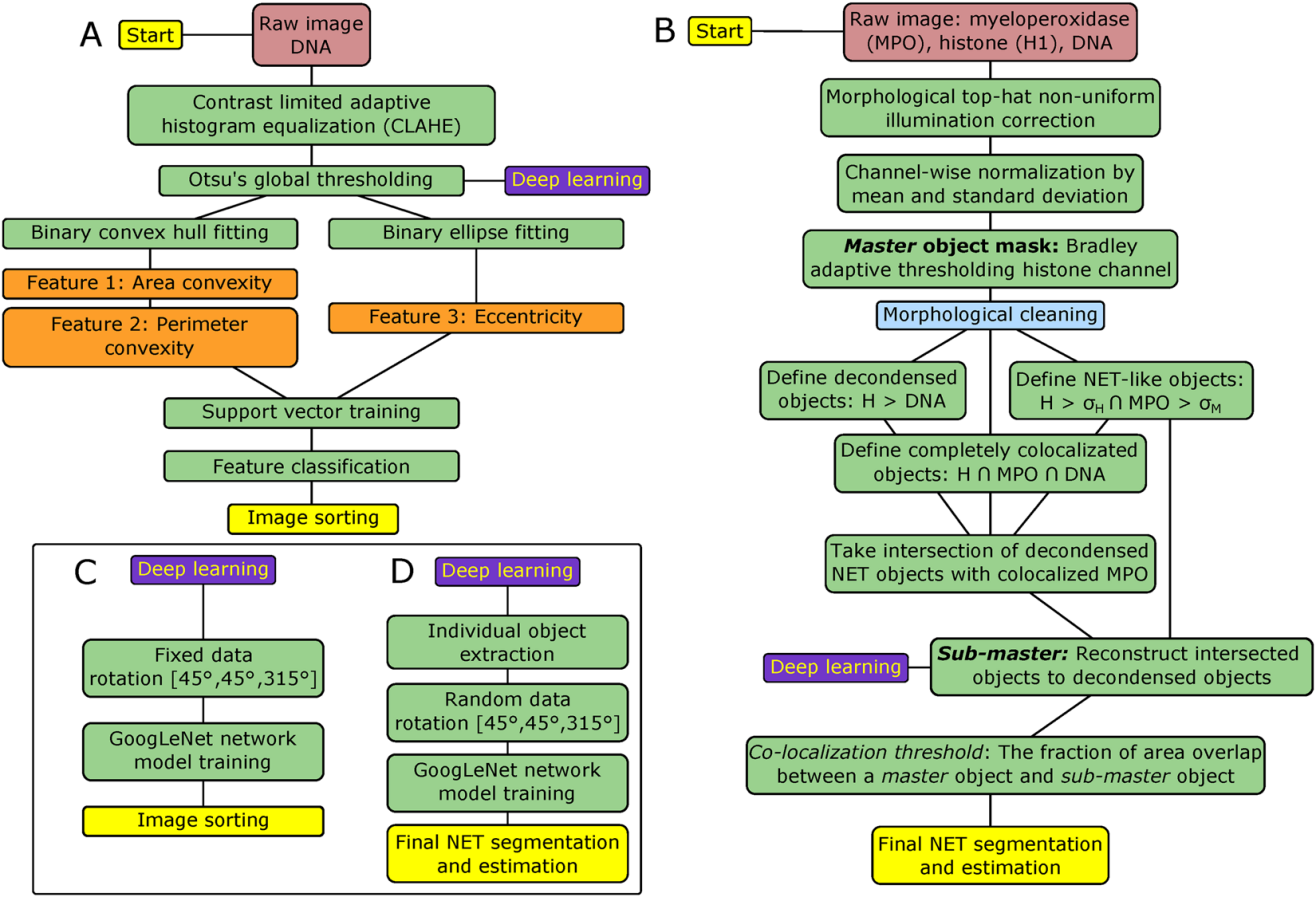
Computational overviews. (**A**) Computational pipeline to classify *in vitro* flow cytometry neutrophil images using a feature extraction and support vector machine (SVM) method. Briefly, images are pre-processed, masked, morphological features are extracted, and subsequently classified via SVM. (**B**) Computational overview of the proposed unsupervised NET segmentation pipeline for thin tissue sections. The NET images are processed for noise, and are further delineated by the co-localization of high levels of histone and myeloperoxidase (MPO). The classification decision for each object in the image is decided by a threshold, primarily dictated by the percent of the object’s area that includes high levels of co-localized MPO and histone. (**C**) An alternative deep CNN method for processing the ImageStream^®^ images. (**D**) An alternative deep CNN method for analyzing thin tissue section images using the object masks developed in Fig. 6B.

Figures 6A and C describe the *in vitro* computation. First, image contrast is adapted using CLAHE^28^, specifically, an exponential distribution with rate parameter *α* = 0.01 is chosen to enhance dim image shadows over bright regions. Images are thresholded either at a fixed value of 10% maximum intensity or based on Otsu’s method^29^. A convex hull and ellipse of the binary region is fit, and used to extract subsequent features^30^. Six total features were examined, and among them, eccentricity, perimeter convexity, and area convexity were best performing. An SVM was trained on top three features with *n* = 1455 neutrophil images, manually annotated, using a Gaussian kernel with scaling factor *σ* = 2 (see Table 4). The other three features examined were found to have minimal impact on classification accuracy or did not provide new unique information to the top three features; see Fig. S3. An alternative approach, Fig. 6C, is to mask out low intensity noise from the images and classify using a deep CNN model.

### *In vivo* thin tissue section preparation

Wild type and NADPH oxidase-deficient mice were administered *A. fumigatus* hyphae, sacrificed, and their lungs were harvested for immunostaining. Primary antibodies directed against MPO and histone H1 were detected with AlexaFluor 568- and 488- conjugated secondary antibodies. DNA was visualized with DAPI. Thin tissue section images were acquired with a 100X oil immersion confocal microscope (Eclipse C1, Nikon Instruments, Melville, NY). These digital images were collected as part of the study conducted by Rohm *et al*.^19^. The animal study followed protocol approved by the Institutional Animal Care and Use Committee at Roswell Park Cancer Institute, and was consistent with federal guidelines and regulations and in accordance with recommendations of the American Veterinary Medical Association guidelines on euthanasia.

### *In vivo* thin tissue section computational pipeline

Figures 6B and 6D describe the *in vivo* computation. Images are first top-hat processed^23^ with a 1.67 µm radius circular structuring element to remove uneven illumination. The pixels in each channel are normalized with respect to the global intensity mean and standard deviation of the respective channel. Next, Bradley local thresholding^22^ with a local window size of 3.3 × 3.3 µm^2^ of the normalized histone channel and a histone brightness threshold of 1% lower than window average is used to identify all possible foreground objects from background (*master* objects). A small morphological disk of radius 0.2 µm is used to clean small objects from the image, and four line structuring elements of length 0.3 µm at orientations 0°, 45°, 90°, and 135° are used to reduce border artifacts and disconnect clustered objects. The resulting objects are further processed for NETs identification. Visually, we detect NETs where there exists high levels of MPO, spatially co-localized with high levels of decondensed histone, and partially spatially co-localized with some amount of DNA. We therefore generate next three auxiliary masks. The first defines any pixel where normalized histone level is greater than the normalized DNA level. The second defines objects which have co-localized levels of both histone and MPO greater than one-unit intensity standard deviation of the respective channels. The third defines objects with histone, MPO, and DNA co-localized. The intersection of these masks is morphologically flood filled^23^ using the second auxiliary mask as reference. This results in *sub-master* objects that are decondensed and highly co-localized for all three markers. Note that this strategy eliminates all the co-localized histone and MPO regions, where histone level is not greater than the DNA level, that are not NETs. Ratio between the *sub-master* component divided by the area of the *master object* is found to be efficient for NET identification (see Fig. 2M). This ratio is the *co-localization level*. Alternatively, one can extract the *sub master* regions along with *master* regions generated in this approach, and classify the objects with a deep CNN.

### Image mean NET co-localization estimator (IMNCE)

The *co-localization threshold* is the *co-localization level* above which objects are labeled NETs. Let us denote the *j* ^th^ object’s *co-localization level* as *l*_*j*_ in a given image at an arbitrary threshold *τ*_*i*_. We estimate the neutrophil extracelluar trap (NET) coverage, *P*_*i*_, as the sum of area of objects with *l*_*j*_ > *τ*_*i*_, divided by the total image area. IMNCE was derived by first incrementing *τ*_*i*_ in 100 steps such that *τ*_*i*+1_ = *τ*_*i*_ + 0.01, ∀*τ* ∈ (0,1]. We define IMNCE to be *E*[{*P*_*i*_}], where *E*(·) denotes the expected value of $\{{P}_{i}\}$.

### Training deep CNNs

Training and testing of both CNNs developed for this work were performed using Caffe^31^, using DIGITs web wrapper (NVIDIA, Santa Clara, CA). We trained our networks using two GPUs, an NVIDIA GeForce 1080 and Titan X Pascale. The GoogLeNet architecture^32^ was used because it was the highest performing of the three (LeNet, AlexNet, and GoogLeNet) available in NVCaffe (NVIDIA’s version of Caffe). In Fig. S4, we compare LeNet, AlexNet, and GoogLeNet performance for both *in vitro* and *in vivo* thin tissue section experiments. GoogLeNet is additionally regularized with dropout layer with 70% ratio of dropped outputs to guard against overfit^32^. Both the networks (one for the *in vitro* images and the other for thin tissue section images) were trained for maximum 50 epochs. A stochastic gradient descent solver was selected with base learning rate 0.01; further, the learning policy was specified to decrease by a factor of ten for each 16 epochs (32% of total training epochs). The pixel-wise mean was subtracted from each image of both datasets of both experiments. All other parameters were left to the default options specified by NVIDIA Caffe. *In vitro* training took ~8.5 mins, and *in vivo* training took ~20 mins.

The GoogLeNet architecture accepts a fixed image size of 256 × 256. To bypass this limitation, input images were padded to the smallest multiple of 256 × 256 which preserves the aspect ratio of the original image, padded with zeros such that each image becomes a square, and the images are subsequently down sampled to size 256 × 256 using bilinear interpolation. This operation was conducted using the “fill” configuration of the DIGITs web wrapper.

For the *in vitro* image analysis, each flow cytometer image was resized as described above. We split the *n* *=* 1,455 images into *n* *=* 908/402/145 images for training/validation/testing. Each *in vitro* object fed to the CNN was the raw DNA image, upon removing noise from the image by eliminating pixels with lower 10% intensity levels in the image. The *in vitro* training data set was augmented by rotating on fixed intervals of 45^0^, from 45^0^ to 315^0^.

For the lung section image analysis, all object patches (*master* and *sub-master* objects) of a given immunofluorescence image were extracted using our unsupervised method (Figs 2, 6B and D), and subsequently resized as discussed above. We split *n* *=* 14 images into *n* *=* 8/4/2 images for training/validation/testing. All objects were extracted resulting in *n* *=* 1,735/969/631 objects for training/validation/testing. These object images and raw image information were used to derive pseudo RGB images to feed the CNN. The first channel of such RGB image is the binary version of the *sub-master* object images. The second is the normalized intensity version of the *master* object images. The third is the binary version of the image with regions of co-localized MPO and histone, with respective intensities greater than one-unit intensity standard deviation of the respective channels, where histone level is greater than the DNA level. The third channel here considers same image regions as in the first two channels. The objects in this pseudo RGB image should contain all possible NETs and non-NETs objects, and when classified using ground-truth labels based on *master* objects, distinguish NETs from non-NETs automatically using deep CNN, without using any threshold parameter as needed for the unsupervised method. The *in vivo* dataset was rotated using a similar strategy to the *in vitro* experiment for data augmentation, except each rotation angle is multiplied by a random scalar between 0 and 1 to produce random rotations. Fixed rotations would not work here because the positive and negative classes were highly imbalanced, and many non-repeated copies of the minority class needed to be created. NET objects were sampled five times as often as negative samples to generate the final training dataset, which resulted in improved recognition of the minority class (holdout sensitivity increased from 0.82 to 0.86).

### Data availability

All of the source code and images used to derive the results presented within this manuscript are made freely available to the public in accordance with Scientific Report’s data availability requirements. Source code and images used to derive the results are available at https://goo.gl/VgXZRs.

## Electronic supplementary material

Supplementary document
