# Supplementary material for: Computational detection and quantification of human and mouse neutrophil extracellular traps in flow cytometry and confocal microscopy
Source: Sci Rep. 2017 Dec 19;7:17755. doi: 10.1038/s41598-017-18099-y (PMC5736696; doi:10.1038/s41598-017-18099-y)
Supplement: Supplementary file 1 — Supplementary document [file 41598_2017_18099_MOESM1_ESM.pdf]

Computational detection and quantification of human and mouse neutrophil extracellular traps in flow cytometry and confocal microscopy

Brandon G. Ginley, Tiffany Emmons, Brendon Lutnick, Constantin F. Urban, Brahm H. Segal, and Pinaki Sarder

Data heterogeneity: *In vitro* NETs

Though most neutrophil images present with morphology like those presented in Figs. 2A & F, like all biological experiments, there are some cases which do not adhere to the norm. Figs. S1A-C show some of the more varied morphologies displayed by *in vitro* NETs. Figs. S1D-E show further examples of negative cases with varied morphology, such as the segmented nucleus of the neutrophil shown in Fig. S1E.

Data heterogeneity: In vivo NETs

The morphological diversity which *in vivo* NETs encompasses is basically unrestricted, as they are not bound by any membrane, having been ejected from neutrophils into the extracellular space. Therefore, they have no defined shape, and represent differently in each unique scenario. Figs. S2-A, C, E, G, I, and K show other examples of fluorescent images of *in vivo* NETs, and Figs. S2-B ,D, F, H, J, and L show their respective transformations under the unsupervised pipeline proposed in this work. Note in Fig. S2H, bronchial epithelial cells mimic the distribution of fluorescence markers found within NETs, with a different spatial distribution circular, which increases the difficulty of the classification task.

Feature space in classifying *in vitro* NETs

We are using six features for *in vitro* NETs classification presented in this article. Fig. S3 depicts the feature space spanned by the optimal three features (Fig. S3A), the three other sub-optimal features (Fig. S3B), as well as a principal projection of the combined feature space (Fig. S3C). The three best features discriminating NETs and intact neutrophils were selected using MATLAB *rankfeatures*.

Comparing LeNet, AlexNet, and GoogLeNet in classifying NETs

The GoogLeNet architecture was used for our convolutional neural network (CNN) based image analysis presented in this article (see the sub-section *Results: Alternate convolutional neural network method*) because it consistently outperforms the two other architectures available in NVIDIA’s version of Caffe (LeNet and AlexNet). In Fig. S4, we compare LeNet, AlexNet, and GoogLeNet performance for both *in vitro* and *in vivo* thin tissue section experiments using deep CNN.

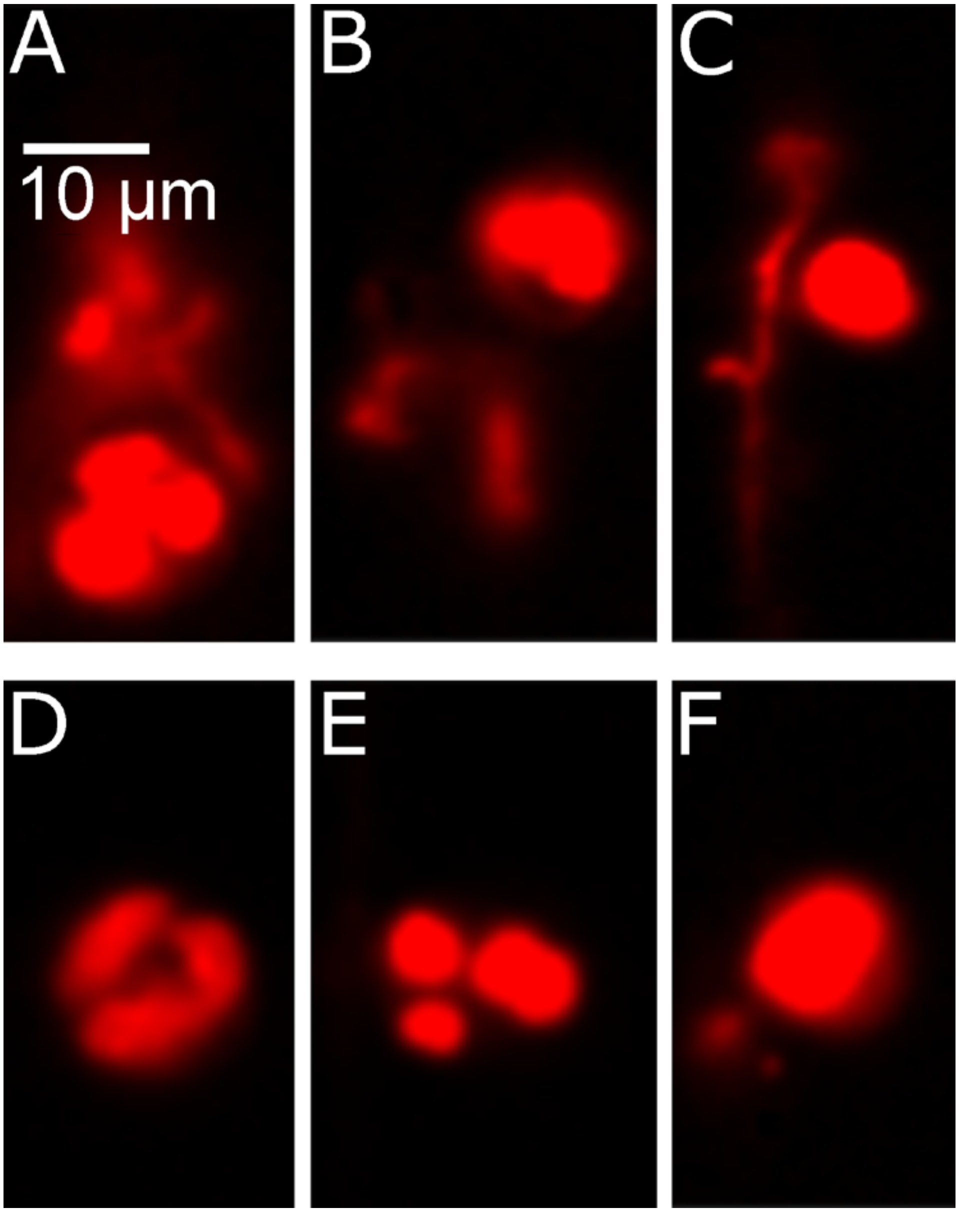

Fig. S1. **Further examples of *in vitro* flow cytometry neutrophil and NET diversity.** (A-C) NET positive images with varied NET morphology. (D-F) NET negative neutrophils with varied morphology, such as the polysegmented nuclei of neutrophil (E).

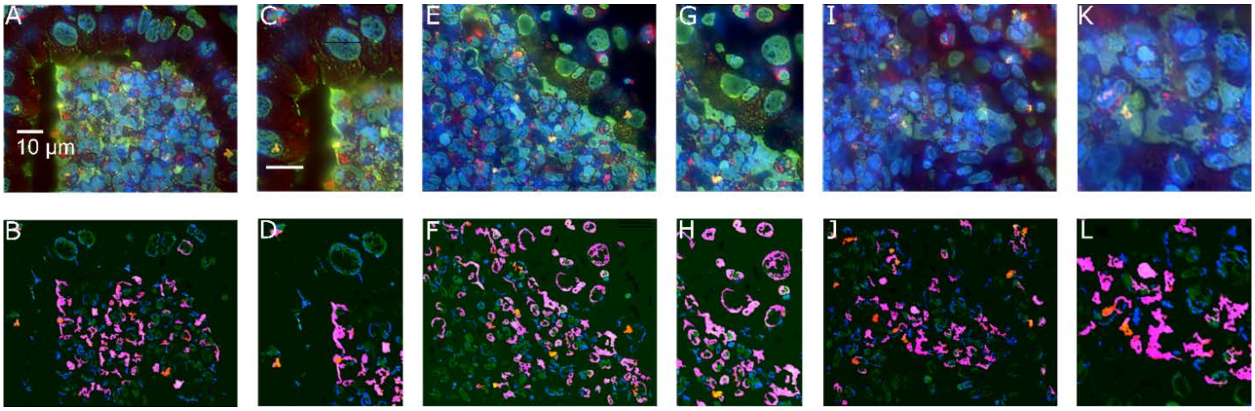

Fig. S2. **Further examples of *in vivo* NET diversity.** (A, E, & I) Example fluorescence full size images, (C, G, & K) show NETs within zoomed sub-sections of each respective image. (B, D, F, H, J, L) show the image directly above itself after transformation under the proposed unsupervised pipeline. (A-D) Exhibition of highly clustered/crowded NETs which complicates segmentation and requires morphological separation. (E-H) Exhibition of the most common false positive, rounded epithelial cells found at the northeast corner of each image. (I-L) Exhibition of exceptionally large NET, which also encompasses live cells and necrotic cells.

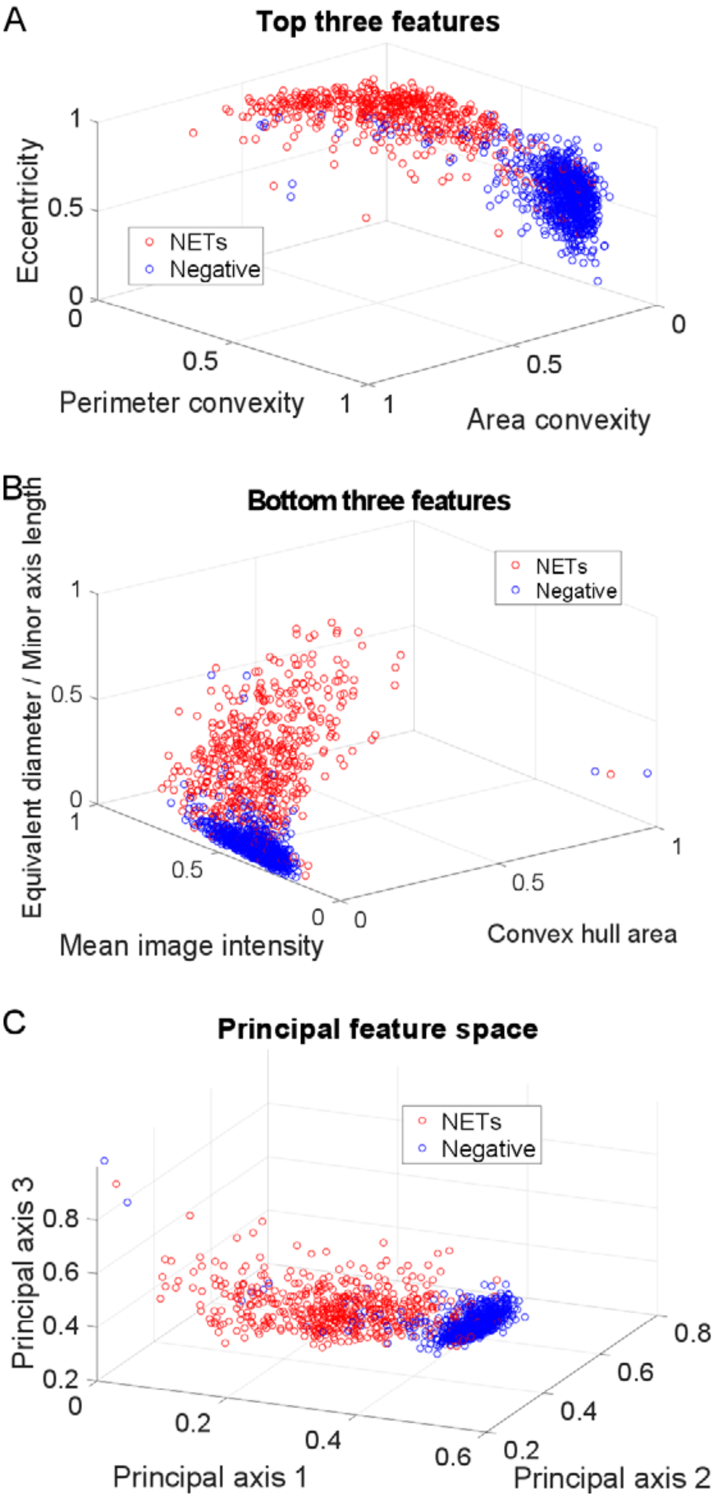

Fig. S3. **Morphological feature spaces of human neutrophils.** (A) Top three of six optimal features used to classify NETs vs neutrophils. (B) Bottom three features examined for classification of neutrophils. (C) Top three principal axes of combined six features.

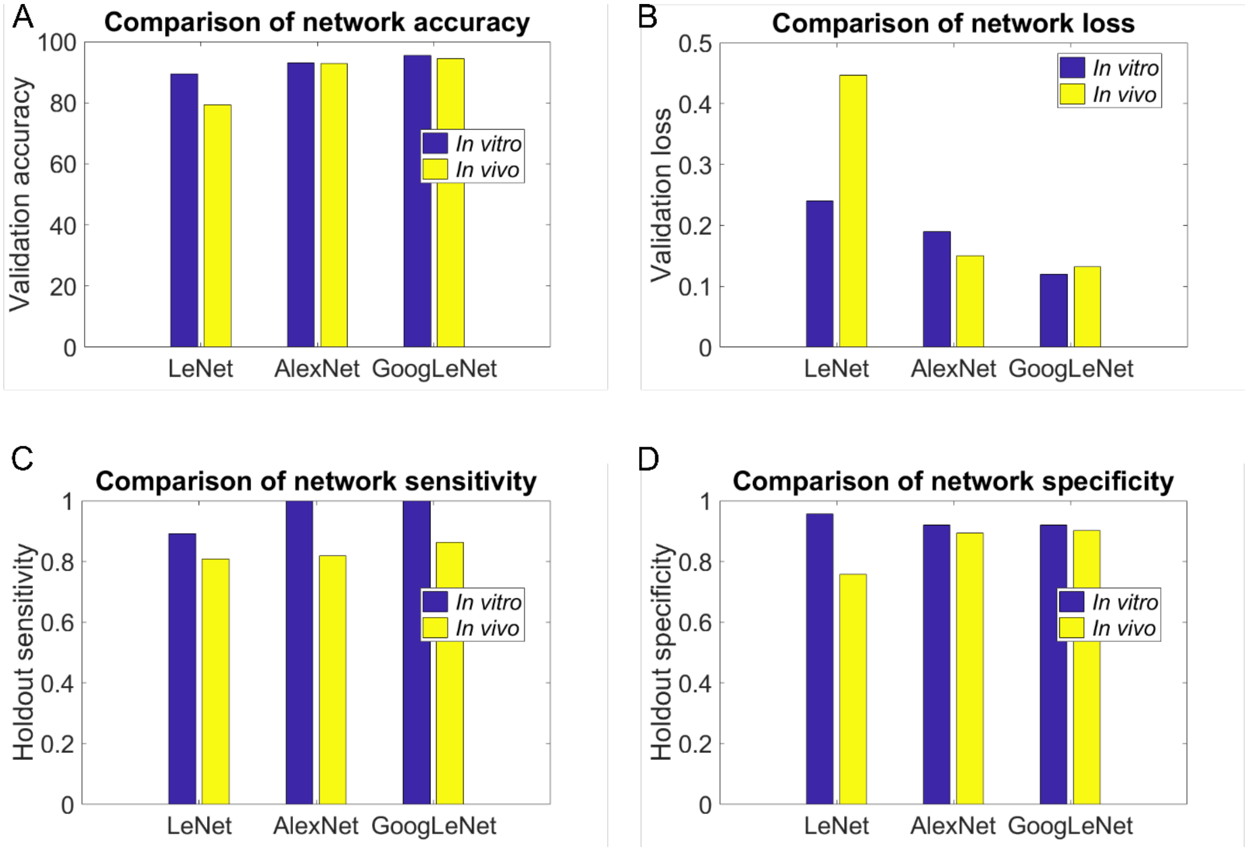

Fig. S4. **Comparison of network architectures implemented by NVIDIA Caffe.** *In vitro* experiments are blue bars and *in vivo* experiments are yellow bars. (A) Validation accuracy for trained networks. (B) Validation loss for trained networks. (C) Holdout sensitivity of each network. Both AlexNet and GoogLeNet demonstrated perfect NET identification at the cost of specificity. (D) Holdout specificity of each network. GoogLeNet performs the best of the three networks on the majority of tests.
